# Overview of the expression patterns and roles of Lipocalin 2 in the reproductive system

**DOI:** 10.3389/fendo.2024.1365602

**Published:** 2024-04-05

**Authors:** Marinela Krizanac, Paola Berenice Mass Sanchez, Ralf Weiskirchen, Sarah K. Schröder

**Affiliations:** Institute of Molecular Pathobiochemistry, Experimental Gene Therapy and Clinical Chemistry (IFMPEGKC), RWTH University Hospital Aachen, Aachen, Germany

**Keywords:** Lipocalin 2 (LCN2), uterocalin, fertility, reproductive system/tract, cancer, estrogen, biomarker

## Abstract

The 25 kDa-sized protein Lipocalin 2 (LCN2) was originally isolated from human neutrophil granulocytes more than 30 years ago. LCN2 is an emerging player in innate immune defense, as it reduces bacterial growth due to its ability to sequester iron-containing bacterial siderophores. On the other hand, LCN2 also serves as a transporter for various hydrophobic substances due to its β-barrel shaped structure. Over the years, LCN2 has been detected in many other cell types including epithelial cells, astrocytes, and hepatocytes. Studies have clearly shown that aberrant expression of LCN2 is associated with a variety of disorders and malignancies, including several diseases of the reproductive system. Furthermore, LCN2 was proposed as a non-invasive prognostic and/or diagnostic biomarker in this context. Although several studies have shed light on the role of LCN2 in various disorders of the female and male reproductive systems, including tumorigenesis, a comprehensive understanding of the physiological function of LCN2 in the reproductive tract is still lacking. However, there is evidence that LCN2 is directly related to fertility, as global depletion of *Lcn2* in mice has a negative effect on their pregnancy rate. Since LCN2 expression can be regulated by steroid hormones, it is not surprising that its expression fluctuates greatly during remodeling processes in the female reproductive tract, especially in the uterus. Well-founded details about the expression and regulation of LCN2 in a healthy reproductive state and also about possible changes during reproductive aging could contribute to a better understanding of LCN2 as a target in various diseases. Therefore, the present review summarizes current knowledge about LCN2 in the reproductive system, including studies in rodents and humans, and discusses changes in LCN2 expression during pathological events. The limited data suggest that LCN2 is expressed and regulated differently in healthy male and female reproductive organs.

## 1 Introduction

Lipocalin 2 (LCN2) is a member of the lipocalin family of proteins known for its specific lipocalin fold that forms a hydrophobic pocket, thus allowing them to act as transport proteins (1). LCN2 was first isolated from human neutrophils and reported as neutrophil gelatinase-associated protein (NGAL) that served as bacterial iron sequester (2). It is believed that its multiple functions probably stem from its ability to act as a monomeric protein (25 kDa), as a homodimer (~46 kDa), and as a heterodimer (~135 kDa) in a complex with matrix metalloproteinase 9 (MMP9) (3). Importantly, only human LCN2 can form dimers (4), whereas murine LCN2 lacks Cys^87^ which is required for covalent disulfide bond formation (5).

Due to its pleiotropic nature, LCN2 was rediscovered and given several names, some of which being oncogene 24p3, siderocalin, p25, α2-microglobulin-related protein and migration-stimulating factor inhibitor (MSFI) (6–8). The protein was given the name siderocalin because LCN2 has the ability to reduce bacterial growth, as it can bind iron indirectly via bacterial siderophores (6, 9). In addition to its physiological role in host immune defense, aberrant expression of LCN2 has been observed under various pathophysiological conditions (10–12).

A role of LCN2 in reproductive tissue was suspected early on after its discovery. Because LCN2 was found to be strongly expressed in the uterine tissue of mice, the term ‘uterocalin’ for LCN2 was introduced (13). However, there are relatively few historical studies in the area of LCN2 in the reproductive system. Even less data is available on the molecular mechanisms by which LCN2 mediates signal transduction in the healthy reproductive system. Currently, three different putative LCN2 receptors, namely soluble carrier family 22 member 17 (SLC22A17), megalin and melanocortin-4 receptor (MC4R), have been described and comprehensively reviewed in (14). The role of these receptors in the reproductive tract is still poorly understood. However, there is evidence that sex hormones influence the expression of LCN2 in mice and humans (15–17).

With this review, our goal was to summarize the available knowledge on LCN2 in female and male reproductive organs. The focus was mainly on the female reproductive tract (uterus, vagina, and ovary). However, in the male reproductive tract, we concentrated on the expression of LCN2 in the testes and epididymis, as its expression in healthy and diseased prostates has been previously summarized (10).

## 2 LCN2 in the female reproductive system

### 2.1 Female reproductive tract

The female reproductive system is tightly regulated by hormones and consists of the vagina, the cervix, the uterus, the fallopian tubes, and the ovaries (18). Ovaries produce and secrete steroid hormones and are responsible for the maturation and release of oocytes. Fertilization takes place in the fallopian tubes (also known as oviducts), after which the fertilized oocyte is transported to the uterus for implantation (19). The cervix not only connects the uterus to the vagina, through which sperm reach the site of fertilization and forms the birth canal, but also serves as a barrier to invading pathogens (20).

Although murine models are used for the research of reproductive organs, notable differences can be observed between human and murine reproductive systems. Unlike humans, which possess a pyriform uterus, mice have a bicornuate uterus consisting of uterine horns with multiple implantation sites that open into the uterine junction (21). Compared to the size of the reproductive tract, mice have rather small oviducts (21). Furthermore, the reproductive system of mice is more similar to that of rats than that of primates (22). Moreover, the time of gestation and the number of offspring differ between humans and rodents (22).

### 2.2 LCN2 expression in the female reproductive system

Although the existence of LCN2 in the uterus has been known for more than 25 years (23, 24), only few studies have addressed the expression, function, and signaling pathways of LCN2 in the female reproductive tract. This is likely due to the fact that so far little is known about the interaction of LCN2 with its various receptors as previously mentioned (14). In the following section, we summarize the studies on LCN2 expression in the female reproductive tract in chronological order.

Interestingly, the first reports of *Lcn2* mRNA (then referred to as 24p3) in the murine female reproductive system were made almost at the same time by two different research groups in 1995 in the USA. Using Northern Blot analysis, Kasik and Rice (24) found high levels of *Lcn2* in the glandular uterine epithelium of pregnant mice during gestation and shortly before giving birth. Liu and Nilsen-Hamilton investigated the expression of *Lcn2* in a turpentine-induced mouse model of Acute Phase Response (APR) as well via Northern Blot analysis (23). In addition to a massive induction of *Lcn2* in the liver during APR, a strongly enhanced expression of *Lcn2* in murine uterine tissue under the same conditions was found (23). Interestingly, the Northern Blot analysis demonstrated that *Lcn2* was not present in the placenta or fetus (23). Further experiments showed that *Lcn2* is also up-regulated during normal pregnancy independently of APR (23). Two years later, the same research group from Iowa State University conducted another study on the expression of LCN2 protein and mRNA in the mice (13). In contrast to increased LCN2 expression in the uterus during APR, Western blot analysis showed no protein expression of LCN2 in the bloodstream or in the amniotic fluid during pregnancy (13). Instead, immunostaining identified the luminal and glandular uterine epithelium as the site of LCN2 protein synthesis (13). Interestingly, the expression levels of *Lcn2* in the liver of the mice did not change during pregnancy, as analyzed by Northern blot analysis (13). It was particularly emphasized that the amount of LCN2 in the uterus during pregnancy and at birth was much higher than the increase detected in the liver during APR. This led to the assumption that LCN2 is involved in local inflammatory processes at parturition (13). In this context, a review article also proposed the theory that LCN2 is subject to separate, local regulation within different tissue types (25).

In 1996, Chu and co-workers demonstrated by Northern blot analysis that *Lcn2* is strongly expressed in the uterus and vagina of adult mice, but not in ovarian tissue (26). Additionally, Western blot analysis revealed that LCN2 was not only detected in uterine tissue, but has also been reported as a component of uterine luminal fluid, secretions that are produced by uterine epithelial cells during estrus cycles in mature rodents (26). Furthermore, their analysis of glycopeptide linkage revealed evidence for *N*-linked but not O-linked glycosylation in LCN2. Thus, two potential sites for *N*-linked glycosylation at Asn^81^ and Asn^85^ of LCN2 can be derived from the primary structure (26).

The key finding of Huang and co-workers in 1999 was that uterine protein and mRNA expression of LCN2 (detected via Western and Northern blot analysis and immunohistochemical staining) is strongly dependent on natural hormonal fluctuations during the estrus cycle in mice (27). In line with this, a study by Burns et al. also shows that *Lcn2* is regulated by hormonal influences in the murine ovary (28). *In situ* hybridization demonstrated that there was no detectable expression of *Lcn2* in the wild type (WT) murine ovary, but depletion of the follicle-stimulating hormone receptor (FSHR) led to greatly increased *Lcn2* levels (28).

In addition to the small number of studies of LCN2 in mice (**Table 1**), there are also few studies on its expression in human tissue. In a study analyzing 50 tissues of human origin by RNA dot-blot hybridization, the uterine tissue was positive for *LCN2* (39). In their comprehensive study, Friedl and co-workers (40) used immunohistochemistry to examine various healthy human tissues for LCN2 protein expression. These results of the study report that LCN2 expression was not found in the female reproductive tract (ovaries, fallopian tube, endometrial glands or stroma, myometrium, uterine cervix). Unfortunately, not all images of the staining results from the screening are displayed in the publication, but the results of the staining have been summarized in tabular form.

**Table 1:** Expression pattern of LCN2 in murine female and male reproductive tract.

Organ	Expression	Detection method	Localization	Reference
Vagina	+++	Northern blot	NA^1^	(26)
Uterus	+++	Northern blot	NA^2^	(24)
	+++	Northern blot	NA^3^	(23)
	+++	Northern blot, IHC^a^	luminal epithelium (day 19 of pregnancy) and glandular epithelium (postpartum)	(13)
	+++	Northern blot	NA^1^	(26)
	+++	Northern blot, Western blot^b^, IHC^c^	luminal and glandular epithelial cells of the endometrium	(27)
	+++	Western blot^d^	NA^1^	(29)
	+++	Northern blot	NA^4^	(25)
	+++	Western blot^e^	uterotubular junction	(30)
	++	IHC^f^	luminal and glandular epithelial cells	(31)
Ovary	–	Northern blot	NA^1^	(26)
	+	RT-qPCR, Western blot^g^, IHC^f^, IF^f^	*Corpus luteum* ^5^	(31)
Testis	–	Northern blot	NA^1^	(26)
	+/-	Northern blot, RT-PCR	Germ and somatic cell fraction^6^	(32)
	+/-	PCR, Southern blot, Western blot^h^	Sertoli cells^7^ and spermatogonial cells^8^	(33)
	++	Western Blot^i^, RT-PCR	Leydig cells	(34)
	++	*In situ* hybridization, Western blot^j^	Leydig cells	(35)
	++	*In situ* hybridization	Leydig cells	(36)
	++	RT-qPCR, Western blot^g^, IHC^f^, IF^f^	Leydig cells	(31)
Epididymis	+++	Northern blot	NA^1^	(26)
	+++	Northern blot, Western blot^k^	epithelial cells and epididymal lumen	(37)
	+++	Western blot	caput epididymis	(38)
Prostate	–	Northern blot	NA^1^	(26)

IF, immunofluorescence; NA, not applicable; IHC, immunohistochemistry; ^1^whole tissue lysates were used; ^2^the whole uterus at the time of birth was analyzed; ^3^the whole uterus of pregnant females was analyzed; ^4^postpartum involuting uterus; ^5^no cell type confirmed but single cells in the *Corpus luteum* were positive in IHC; whole tissue lysate was used for Western Blot and RT-qPCR. ^6^Northern blot analysis of wild type testis showed no expression of *Lcn2* (at different ages), but expression of *Lcn2* was detected by RT-PCR in testicular gonadal cells (isolated via fluorescence-activated cell sorting) from 13.5-dpc and 3-week-old wild type mice in germ cells and the somatic fraction; ^7^a barely detectable level was found in primary Sertoli cells alone (isolated from 7-day-old mice), but a strong expression was observed in coculture with spermatogonial cells and after treatment with spermatogonial-conditioned medium; ^8^defined as the c-Kit^+^ cell fraction. ^a^fixation in 2% paraformaldehyde, antibody: self-made in rabbits; ^b^self-made rabbit antibody; ^c^fixation in Bouin’s solution, self-made rabbit antibody; ^d^self-made rabbit antibody; ^e^rabbit polyclonal anti-LCN2 antibody (#sc-50351, Santa Cruz Biotechnology); ^f^fixation in 4% neutral buffered formaldehyde (stabilized with methanol), polyclonal goat anti-LCN2 antibody (#AF3508; R&D Systems; 1:40 for IHC); ^g^polyclonal goat anti-LCN2 antibody (#AF3508; R&D Systems; 1:800); ^h^polyclonal goat anti-LCN2 antibody (#AF1857, R&D Systems); ^i^monoclonal rat anti-LCN2 antibody (#MAB1857, R&D Systems; 1 µg/ml); ^j^anti-LCN2 antibody (no product number indicated, Abcam, 1:1000); ^k^self-made in rabbit antibody. Symbols mean: -, no expression; +/-, very low expression; +, low expression; ++, moderate expression; +++, strong expression.

According to Human Protein Atlas (https://www.proteinatlas.org/, last accessed on 21.3.2024), high expression of *LCN2* mRNA was detected in the vagina and cervix, while protein expression was proven only in the cervix. Low or no LCN2 expression was detected in the ovary, endometrium, fallopian tube, and placenta (**Table 2**). There are no data on the tissue expression of human LCN2 in the vagina, but it was found via enzyme-linked immunosorbent assay that LCN2 is secreted into the vaginal fluid and participates in vaginal immune response (41).

**Table 2:** Summary of LCN2 mRNA and protein expression according to Human Protein Atlas*.

Organ	IHC		RNA seq	
	Expression	Localization	Expression	Localization
Vagina	–	NA	+	NA
Cervix	+++	glandular cells	+++	Smooth muscle cells, squamous epithelial cells, glandular cells
Uterus	–	NA	+	Smooth muscle cells, stromal cells, glandular cells
Oviduct	–	NA	–	NA
Ovary	–	NA	+	Smooth muscle cells, stromal cells
Testis	–	NA	+	Cells in seminiferous ducts
Epididymis	–	NA	+	Smooth muscle cells, glandular cells
Prostate	–	NA	+	Glandular cells, smooth muscle cells

*All data was taken from the Human Protein Atlas [https://www.proteinatlas.org/ENSG00000148346-LCN2, last accessed on 21.3.2024]. IHC, immunohistochemistry; NA, not applicable; IHC, immunohistochemistry; RNA seq, RNA sequencing. Symbols mean: -, no expression; +, low expression; +++, strong expression.

A more recent study by De La Chesnaye and colleagues showed for the first time ovarian expression of LCN2 in rats (42). RT-qPCR showed that ovarian *Lcn2* mRNA increased from date of birth to 30 days (pre-pubertal period) in rats (42). Western blot analysis showed that adult rats (without definite age mentioned) show stronger expression of LCN2 protein in ovaries than testes (42). Furthermore, cellular localization of LCN2 via immunostaining detected positive signal in granulosa cells (of primordial and growing follicles), in the oocyte, in the zona pellucida and in the antrum of developed follicles of adult rat ovaries (42).

**Figure 1:**
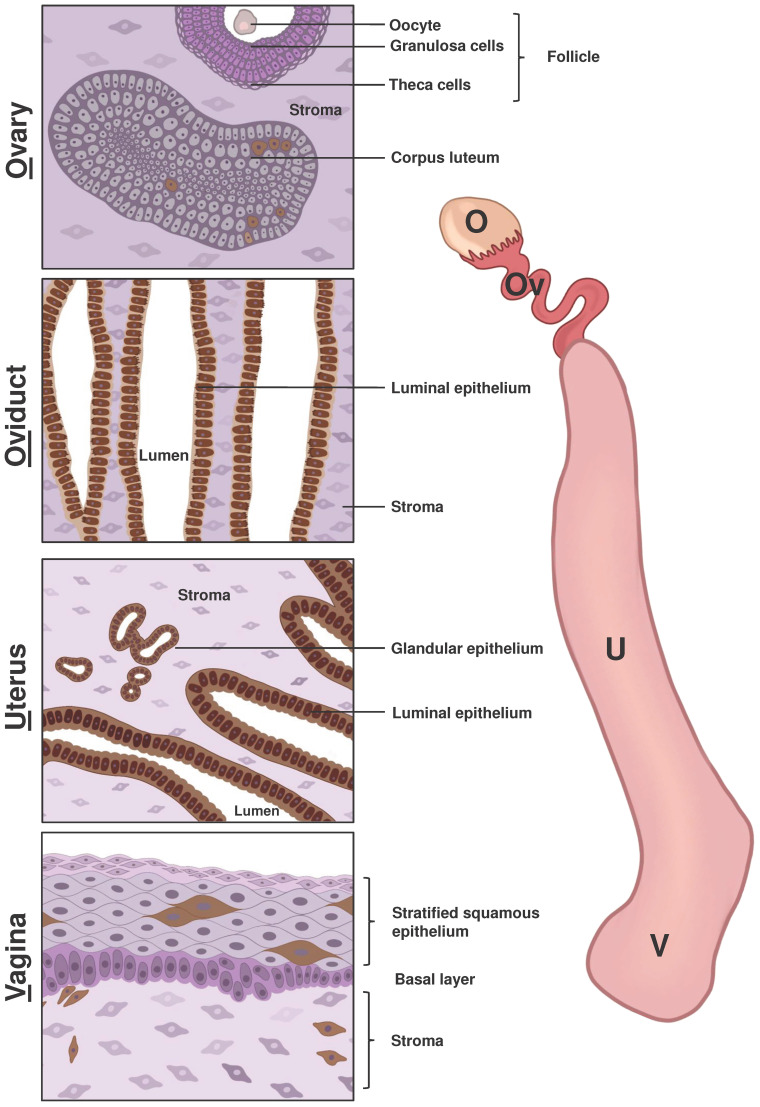
Schematic representation of LCN2 expression in the main components of the female reproductive system. The right side of the figure displays the primary compartments of the mouse female reproductive tract, including the ovary (O), oviduct (Ov), uterus (U), and vagina (V). The left side of the figure depicts a scheme of immunohistochemical staining of LCN2 in different parts of the female reproductive system based on previous research and our own findings using the anti-LCN2 antibody AF3508 from R&D Systems. Cells shown in shades of pink and purple indicated a lack of LCN2 expression, while cells with brown staining indicate positive LCN2 expression. The ovarian stroma consists of epithelial cells, fibroblast-like cells and smooth muscle cells. Growing ovarian follicles contain various cell types, including oocytes surrounded by granulosa and theca cells. Only cells in the *Corpus luteum*, which form from cells remaining in the pre-ovulatory follicle, were positive for LCN2. In both the oviduct and uterus, the luminal epithelium displayed positive staining for LCN2, while stromal cells remained unstained. Additionally, the glandular uterine epithelium showed positive LCN2 expression. In vaginal tissue, positive LCN2 staining was observed in several cells of the surface epithelium and stroma (*lamina propria*).

The most recent study by our research group showed contrasting results for the expression of LCN2 in the ovary in mice, compared to the study described previously in rats. We found low but detectable mRNA and protein expression of LCN2 in the ovaries of B6N(Cg)-Esr1^tm4.2Ksk^/J mice through immunohistochemistry, Western blot, and RT-qPCR analysis (31, 43). The study revealed a correlation of increased LCN2 expression and estrogen receptor alpha (ERα, *Esr1*) deficiency in adult murine ovaries (31). In addition, in adult WT animals, the previously described estrus-dependent expression of LCN2 was confirmed in luminal and glandular uterine epithelium via immunohistochemical stainings (31). We have recently shown that LCN2 is also strongly expressed in parts of the oviduct and in the vagina of adult WT animals (**Figure 1**). Importantly, a subsequent study showed that the ovaries of *Esr1*-deficient mice exhibited iron accumulation, increased levels of LCN2, and signs of aging (43).

Limited data suggest that there are differences in LCN2 activities in mice (summarized in **Table 1**) and humans (summarized in **Table 2**), which may be related to the aforementioned ability of human LCN2 to form dimers. Furthermore, taking all reports into account, the conclusion can be drawn that LCN2, which is usually viewed as a systemic inflammation indicator, is regulated by specific local mechanisms within reproductive tissue without affecting the rest of the organism. Unfortunately, mechanistic insights into the mechanism of action of LCN2 in the female reproductive tract are still scarce. A study by Lee and co-workers suggested that post-translational modification of LCN2 may play a role in its biological function, as there is a phosphorylation site of protein kinase C at Ser^88^ in murine uterine protein (29).

We summarized the current knowledge on the expression of murine LCN2 in different compartments of the female reproductive tract in **Figure 1** and included findings of our own (unpublished) immunohistochemical LCN2 stainings. Further comprehensive studies are needed to determine the precise cell type expressing LCN2, for instance, in the ovary, oviduct, and vagina and whether they are subject to hormonal fluctuations of the estrus cycle.

### 2.3 LCN2 in fertility, pregnancy, and involution

Throughout history, the expression of LCN2 in the reproductive system has been studied primarily in the context of pregnancy and reproduction. Studies gave evidence that LCN2 levels fluctuate during murine estrus cycle (27, 31). The murine estrus cycle consists of 4 phases, namely proestrus, estrus, metestrus, and diestrus which repeat every 4 to 5 days unless interrupted by pregnancy, pseudopregnancy, or anestrus (44). As already briefly mentioned, Huang et al. showed that the level of *Lcn2* mRNA was high in the proestrus and estrus, then declined dramatically from the metestrus to diestrus in the uterus (27). Consistent with this observation, the LCN2 protein was abundant in the proestrus, decreased from estrus to metestrus, and declined to a very low level in the diestrus. We confirmed this finding and further showed that when hormone balance is disrupted by depletion of *Esr1*, the corresponding *Esr1*-deficient mice remain permanently in the diestrus (31). This is accompanied by a constant and massively reduced expression of LCN2 in uterine epithelium, which was observed in immunohistochemical staining (31).

Most existing publications studied LCN2 in the context of gestation, parturition or postpartum (13, 23, 24, 26). Liu and colleagues analyzed tissues stained with anti-LCN2 antiserum from mice on day 19 of pregnancy and 1 day postpartum (13). LCN2 was detectable in the luminal uterine epithelium (day 19). On day 1 after delivery, it was also found in the glandular epithelium. Kasik and Rice speculated that uterine *Lcn2* mRNA (around birth) could be due to neutrophils in the uterus or stem from different cell types (such as myometrial cells) (24).

Following birth, the uterus undergoes a process called involution, whose goal is to return the uterus to its prepregnancy state. It has been observed that the postpartum involuting uterus is a major site of LCN2 expression (25). The authors speculate that in this context, LCN2 serves as an inducer of neutrophilic apoptosis, thus protecting the uterus from oxidative stress and carcinogenesis during the remodeling phase.

Others studied LCN2 in uterine tissue of rats (45) in context of pregnancy and involution. Western blot analysis show that LCN2 was strongly elevated on day 22 of pregnancy and on the first two days after delivery compared to non-pregnant uterine tissue. In pregnant and non-pregnant rats (in the proestrus), uterine LCN2 expression was restricted to the uterine luminal and glandular epithelium of the uterine and did not colocalize with Myeloperoxidase (detected via immunostaining), which is also up-regulated during postpartum involution but in different cellular compartments (in the vessel-rich layer and in the endometrial stroma).

The uterine tissue is also involution, as is the mammary gland. Northern blot analysis demonstrate that *Lcn2* expression was barely detectable in the mammary gland of virgin, pregnant or lactating mice (46). In contrast, the involution process that induced strong tissue remodeling of the mouse mammary gland started with weaning and was accompanied by strong expression of *Lcn2* mRNA and protein (46, 47). *In situ* hybridization demonstrated that *Lcn2* expression was restricted to epithelial cells of mammary gland alveoli (46). Mechanistically, Bong and colleagues found that overexpression of *Lcn2* drives mammary epithelial cells to apoptosis. Interestingly Western blot analysis show that the LCN2 protein is secreted from epithelium into the milk of lactating female mice, indicating an important function for the pups as well (47). Not less important, it was also speculate that *Lcn2* and its receptor (Slc22a17 alias 24p3R) are involved in apoptotic signaling in the perinatal ovary, where numerous ovaries are lost through apoptosis (42).

*LCN2* has been reported to play a role during pregnancy not only in rodents, but also in mares, where *in situ* hybridization showed that it was localized in the equine uterine glandular epithelium (48). Immunohistochemical stainings demonstrate that LCN2 protein is expressed in gravid endometrium and secreted into the luminal fluid (48). Therefore, it was speculated that LCN2 could function as a carrier for small substances from the mother to the fetus (48).

Several reports implicate LCN2 in the fertilization process (30, 49). A study by Watanabe and colleagues identified LCN2 produced by the female mouse reproductive tract as a sperm-capacitating agent that alters the membrane properties of sperm in preparation for fertilization (30). More specifically, LCN2 levels reach their peak during estrus at uterotubular junction, where sperm maturation is believed to begin, forcing the sperm to undergo lipid raft reorganization, cholesterol efflux, and glycosylphosphatidylinositol-anchored protein shedding necessary for fertilization. Furthermore, the same study reported that LCN2 has the ability to increase murine *in vitro* fertilization efficiency, making it a great target to study for clinical application.

On the other hand, other studies reported a negative correlation between LCN2 and fertilization (50, 51). More precisely, with the use of computer-assisted sperm analysis method, cytochemical staining and detection of the protein tyrosine phosphorylation pattern Lee and co-workers reported that the LCN2 protein play a role in stimulating flagellar motility but preventing acrosome reaction. The same group confirmed previous indications of the inhibitory effect of LCN2 on *in vitro* fertilization (51). To be precise, the presence of LCN2 reduced the response of sperm to bovine serum albumin (BSA) and calcium by suppressing intracellular pH elevation, calcium uptake, cAMP accumulation, and protein tyrosine phosphorylation of BSA/calcium-stimulated sperm.

The most important report on the implication of LCN2 in the fertilization process is probably the one that says that Lcn2KO mice show compromised fertility (52). The study reports that although *Lcn2* deficient males appear to be fertile, *Lcn2*-deficient females show a significantly (*p* < 0.05) decreased pregnancy rate compared to WT littermates when crossed to WT or *Lcn2*-deficient males. Taken together, these data indicate that sperm maturation and fertilization are highly dependent on the LCN2 status of the female individual.

## 3 LCN2 in the male reproductive system

### 3.1 Male reproductive tract

The male reproductive system consists of external genitals such as penis, scrotum, and testes and the internal organs, including the vas deferens, urethra, and prostate gland. The interaction of the individual parts is controlled by the hypothalamic-pituitary-gonadal hormone axis and is essential for male fertility. Only a brief overview is given here, since the various processes are very complex and precisely regulated and are explained elsewhere (53, 54). The male reproductive organs have various functions including production of testosterone, spermatogenesis, sperm maturation and –storage, and the release of the sperm. The testes have both an endocrine function (e.g., testosterone production) and an exocrine function (spermatogenesis). Sperm maturation and storage occur in the epididymis. The sperms are transported via the *Vas deferens* to the prostate and urethra. The prostate gland produces the fluid secretions that support the sperms. Both sperm and urine from the bladder are excreted through the urethra to the outside via the penis. It is important to keep in mind that there are various differences in the male reproductive tract between species, such as gross anatomy and histology. Compared to humans, for example, rodents have a multilobed prostate and additional glands (54).

### 3.2 LCN2 expression in the male reproductive system

According to Human Protein Atlas (https://www.proteinatlas.org/, last accessed on 21.3.2024), *Lcn2* mRNA expression was not detected in the testes, epididymis, and seminal vesicles, while only low expression is reported in healthy prostate. The data correspond to the study by Friedl and co-workers who analyzed the expression of the LCN2 in the human prostate gland and found strong expression in glandular foci and a positive signal in epithelial cells (40). Furthermore, RNA dot-blot hybridization with a probe containing the *LCN2* cDNA sequence showed specific expression in human prostate tissue (39). To date, no further data are available on the physiological expression and function of LCN2 in the male human reproductive tract.

In contrast to the limited data available on human reproductive tissues, there are some studies on rodent models that investigated LCN2 in different parts of the male reproductive system. Chronologically, Chu and colleagues in 1996, were the first to mention LCN2 in the male reproductive tract (26). Northern blot analysis of various tissues of adult mice revealed a strong expression of *Lcn2* in the epididymis, but not in the testes, prostate, seminal vesicles, or coagulating glands (26). Four years later, the group confirmed their findings by immunolocalization and Northern blot analysis (37). LCN2 was expressed in epithelial cells and the lumen of mouse epididymis (37). Both the mRNA and the LCN2 protein were already detectable in 2-week-old mice and remained at a constant level during the observed period (up to 12-weeks). Interestingly, a decreasing gradient of LCN2 expression was detected from the caput to the caudal region of the epididymis, as well as its association with sperm. The finding was confirmed by another research group, who detected the presence of LCN2 in the caput region of the epididymis via immunolocalization (38). In another study, it was proposed that LCN2 is responsible for internalization of the protein–ligand complex by spermatozoid cells (55). Most notably, LCN2 has been reported to serve as a physiological mechanism for the delivery of ferric ion to epididymal spermatozoa (55). Taking into account the findings, it was concluded that LCN2 is an important component in spermatozoa processing.

In the initial studies mentioned above on LCN2 in the male reproductive tract, no expression was detected in the testes, but the studies of the following years showed contrasting results. To better classify the ambiguous results, the histological structure and function of the testes are briefly described. Histologically, testes consist of tubular glands covered by the tunica albuginea (54). They comprise testicular tubules (seminiferous tubules), including Sertoli cells and germ cells (spermatogonia, spermatocytes, spermatids) and the interstitial compartment with the endocrine Leydig cells (54). Sertoli cells function as the main cells required for maturation and differentiation of germ cells (54).

Tanaka and colleagues found contrasting results regarding *Lcn2* expression in murine testes. Northern bot analysis showed no expression in wild type mice from 19.5-dpc up to 8 weeks (32). In the same study, *Lcn2* expression was determined in germ cells and somatic cells via RT-PCR after fluorescence-activated cell sorting of testicular gonadal cells from wild type (13.5-dpc and 3-week-old) mice (32). It was assumed that the somatic cells are mainly Sertoli cells, but no Leydig cell markers, which also belong to the somatic cells, were examined (32). A subsequent study by the same group showed, that primary Sertoli cells, obtained from the testes of 7-day-old mice through enzymatic digestion followed by magnetic activated cell sorting, exhibited undetectable levels of *Lcn2* (33). Additionally, they reported that its expression in juvenile primary murine Sertoli cells is highly dependent on germ cells, more specifically spermatogonial cells (33). The freshly isolated primary Sertoli cells were positive for *Lcn2*, but loose expression levels when cultured for 1 week alone but not in coculture with primary spermatogonial cells. Interestingly, *in vitro* treatment of the Sertoli cells with spermatogonial cell-conditioned medium caused nuclear factor-B (NF-κB) pathway mediated transcription of the *Lcn2* gene (33), providing first insights into the signaling mechanisms responsible for LCN2 expression in the testes. Moreover, it was shown that *Lcn2* is regulated in a different manner in testicular cells compared to inflammatory immune cells, as commonly known inducers (Interleukin-1β and lipopolysaccharide) fail to induce *Lcn2* in an immortalized Sertoli-B cell line (33). Furthermore, transcriptional regulation of the *Lcn2* gene in juvenile Sertoli cells was independent of IκBζ (33).

In 2009, it was speculated that artificially generated electromagnetic fields (EMF) affect sperm motility and could affect LCN2 expression in mice (34). This study provides further insights into the expression of LCN2 on mRNA and protein level in healthy testes. Total testicular mRNA, isolated from untreated adult 8-week-old mice, showed strong *Lcn2* expression demonstrated by RT-PCR (34). When mice were exposed to a 3 mT (50 Hz) magnetic field (for 6 days a week and 4 hours a day) for 8 weeks, *Lcn2* mRNA expression and protein levels decreased significantly (34). Mechanistically, it was discussed that LCN2 is involved in EMF-induced apoptosis in germ cells.

Years later, a research group analyzed the pattern of *Lcn2* expression with increasing age in mice and provided the first evidence for *Lcn2* in murine Leydig cells (35). *In situ* hybridization demonstrated that *Lcn2* levels show an age-dependent increase in testes from day 1 to 8 months in Leydig cells, then reduce by the twelfth month, portraying *Lcn2* as a developmentally regulated gene (35). However, while that study detected LCN2 protein expression in Leydig cell, it reported an absence of LCN2 in Sertoli and germ cells (35).

A study by De La Chesnaye and coworkers in 2018 studied LCN2 for the first time in adult rat testes and reported positive expression solely in germ cells (42). In addition, the mRNA expression profile and cellular location of LCN2 were analyzed in gonads collected from fetal rats at 21 days post-coitum, as well as neonatal rats at 0, 2, 4, 6, 12, 20 and 30 postnatal days (42). Interestingly, expression in testes collected at all stages was lower than in ovaries of female rats. In this study, the expression of the LCN2 receptor SLC22A17 was shown in germ cells at different developmental stages (42). On the contrary, a consecutive study done by the same group in 2020, reporting detectable LCN2 expression in Sertoli and Leydig cells (56). Although both publications contain manufacturer information on the antibody used (which was the same), only one of the studies provides information on the catalog number, therefore, it cannot be completely ruled out that different antibodies were used. Unfortunately, this discrepancy is not addressed in the study, so it is not possible to definitively say where exactly LCN2 is expressed in the rat testes.

Recently, studies observed high expression of *Lcn2* in adult Leydig cells via *in situ* hybridization (36). This is in line with the most recent publication by our group studying LCN2 in reproductive tissues. There, moderate expression of *Lcn2* mRNA and protein expression was found in the testes of adult mice (31). In addition, immunolocalization confirmed positive signals in interstitial Leydig cells (31). Furthermore, this study found *Esr1*-dependent expression of LCN2, since it was greatly increased in *Esr1*-deficient testes (31).

**Figure 2:**
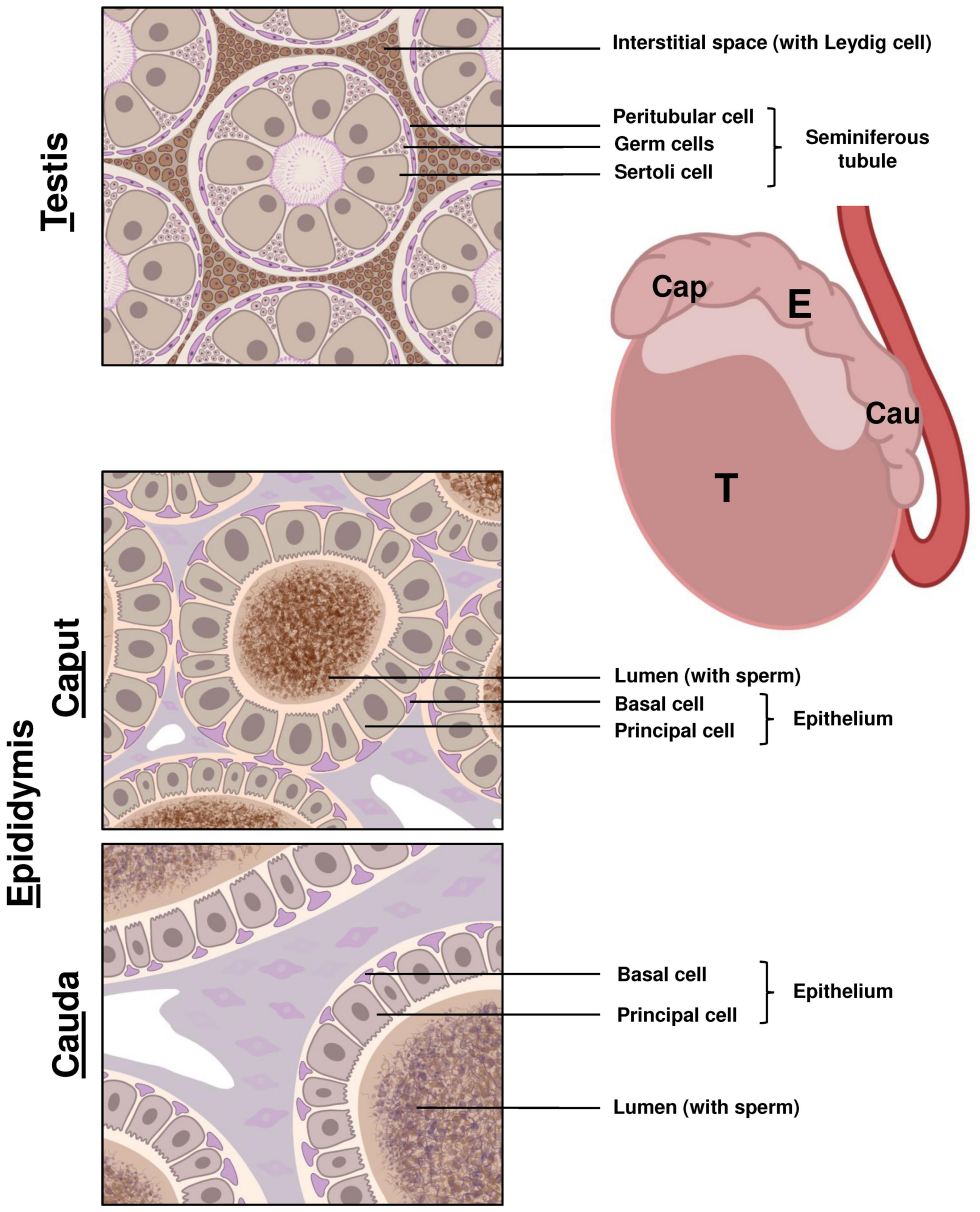
Schematic representation of LCN2 expression in the main components of the male reproductive system. The right side of the figure shows the main components involved in sperm maturation and development in the murine male reproductive tract, namely the testis (T) and epididymis (E) which can be histologically divided into the caput (Cap), corpus, and cauda (Cau) regions. The left side of the figure is a schematic representation of immunohistochemical staining of LCN2 in the different compartments using the anti-LCN2 antibody AF3508 from R&D Systems. Cells shown in shades of pink and purple represent cells negative for LCN2, while cells with brown staining are positive for LCN2. Spermatogenesis begins gradually in the germ cells located in the seminiferous tubules of the testis. Positive staining for LCN2 was only observed in interstitial located, hormone producing Leydig cells. The spermatozoa (sperm), produced in the testis, are transported for maturation and storage to the epididymis. In the caput epididymis, a high expression of LCN2 is visible in the lumen where sperm maturation occurs. A decline in LCN2 expression can be observed when comparing sperm in the lumen of the cauda to the caput epididymis segment.

In summary, it can be concluded that the expression and function of LCN2 in the male reproductive tract are not yet fully understood. Although research suggests that LCN2 plays a vital role in sperm maturation, to understand the exact mechanisms by which LCN2 exerts its functions, a detailed screening of LCN2 expression in different compartments with a well-established antibody (such as anti-LCN2 AF3508 from R&D Systems) would be essential. Therefore, we have schematically summarized the immunohistochemical localization of LCN2 expression in the male reproductive tissues (of mouse testis and epididymis) in **Figure 2** that we have found in unpublished work. It is important to understand which cells express LCN2 and which putative receptors are essential. In this way, the molecular mechanisms of its action can be finally uncovered.

## 4 LCN2 in the pathology of female reproductive tissue

The presented article summarizes that there are only limited, partially ambiguous studies on the physiological expression and function of LCN2 in the reproductive tract. However, there are several studies that focus on LCN2 in different diseases and pathologies of both male and female reproductive tracts. In the following sections, LCN2 is examined in the context of various diseases.

### 4.1 Hormonal imbalance

Steroid hormones, especially estrogens, are essential players in a variety of biological processes in the female reproductive tissue. They enable folliculogenesis, successful ovulation, and mediate proliferation and differentiation of uterine tissue (57). Studies show that the *LCN2* promoter contains an estrogen-responsive element and its expression can be controlled by estrogens (17). If hormone levels are not in balance, hormone deficiency conditions can occur, for example, hypogonadism or during menopause. Since LCN2 is influenced by natural hormonal fluctuations of the estrous cycle (27), it is likely that its expression is affected under such conditions. In a study by Liu and co-workers, the dependence of LCN2 on hormones was investigated using an ovariectomized mouse model, which is a valuable tool for understanding estrogen deficiency (16). *In situ* hybridization and Western blot analysis gave evidence that estrogen is the main hormone for inducing LCN2 levels in uterine tissue (16). On the other hand, deletion of *Esr1* in mice, one of the receptors through which estrogens can mediate signals, does not induce LCN2 in uterine epithelium (16). This is in line with the findings of our recent study using *Esr1*-deficient mice (31). Since the mice in the studies by Liu et al. had their ovaries removed due to the experimental model (16), the authors were unable to make any statement about the expression of LCN2 in the ovary. Kessel et al. showed that the depletion of *Esr1* in female mice leads to a massive increase in LCN2 expression in the ovary (31).

Obesity affects not only LCN2 expression in adipocytes (58), but also in the reproductive tract. A study using rats showed that ovarian LCN2 strongly decreased in pups from obese mothers compared with pups of mothers fed control diet (56). This scenario was as well observed in rat testicles of pups from obese mothers compared with control animals (56). However, the molecular mechanisms of *Lcn2* regulation are poorly understood, but there is a general assumption that SLC22A17 and megalin play essential roles (56). Further studies should aim to understand the molecular mechanisms, which in turn will also allow conclusions to be drawn about the physiological function of LCN2.

### 4.2 Polycystic ovarian syndrome (PCOS)

Hormonal imbalances, including a low level of estrogen and high levels of testosterone, are common in patient with polycystic ovarian syndrome (PCOS) (59). LCN2 is likely involved in PCOS as obesity is linked to this condition (59) and LCN2 is known to be strongly expressed by adipocytes (58). However, there are limited studies on the subject, and existing data is inconclusive. As previously reported, human LCN2 has the ability to form complexes with MMP9 and regulate its function (3). A study conducted on 80 subjects (40 healthy and 40 patients with PCOS) reported lower serum levels of LCN2 as well as LCN2/MMP9 complex in PCOS patients (60). Another study found no difference in serum concentration and activity of LCN2/MMP9 complex between the diseased and healthy groups (61). Sahin and co-workers also found no difference in serum LCN2 levels between patients with PCOS and control patients (62). On the contrary, Cakal and colleagues reported increased serum levels of LCN2 in woman with PCOS (30 patients) compared to body mass index-matched controls (30 woman) (63). There are two studies that report that LCN2 is not affected by PCOS, but that obesity in patients with PCOS is associated with alterations of serum LCN2 levels (64, 65). Loss of weight significantly decreases LCN2 levels in obese/overweight PCOS patients (64, 65). Possible hormonal fluctuations in patients of these studies are a possible reason for the different results, suggesting the notion that hormonal differences might affect LCN2 or activity of the LCN2/MMP9 complex (61). More comprehensive studies on urine, blood, and tissue are needed to understand the role of LCN2 in PCOS.

### 4.3 Issues in pregnancy

Shortly after the discovery of LCN2, it became obvious that it plays a role in fertilization, implantation, and pregnancy in mice (13, 23, 24, 26).

However, LCN2 has also been implicated in pregnancy complications in humans. Meta-analysis comparing women with healthy pregnancy and preeclampsia (PE) detected that high circulating LCN2 is associated with PE, which may be independent of the trimesters for blood sampling and the severity of PE (66). In line, Stepan and colleagues found that maternal LCN2 serum levels increased significantly in PE patients (67).

Based on these findings, it is likely that elevated circulating LCN2 expression is associated with increased vascular and metabolic risk in patients with PE. Furthermore, meta-analysis of data comparing gestational diabetes mellitus (GDM) patients to parturient with normal glucose tolerance identified higher blood LCN2 in GDM patients (68). Proteomics analysis of amniotic fluid obtained from patients diagnosed with a sonographic short cervix (< 25 mm) revealed a proteomic signature of increased risk of imminent delivery containing increased LCN2 protein (69).

### 4.4 Infection and inflammation

During early pregnancy, stimulation of uterine tissue with lipopolysaccharide or injection of *Escherichia coli* leads to a strong induction of LCN2 (16). In addition, LCN2 is secreted in the vaginal fluid and is involved in the vaginal immune response (41). There is a positive correlation between the increase in leukocyte infiltration and the level of LCN2 in the vaginal fluid of woman suffering from vaginal inflammation (70). Interestingly, LCN2 expression was found to be increased during vulvovaginal candidiasis infection, but decreased in bacterial vaginosis, two common vaginal disorders in reproductively aged women (41). The differences may be related to different microbiota composition in the different disorders and the authors speculate that low levels of LCN2 might be favorable for bacterial processes that use siderophores to obtain iron for proliferation (41). Another study in connection with group B *Staphylococcus* (GBS) infections during pregnancy assumes that LCN2 prevents iron uptake by GBS and thus restrict their growth (71). The immune defense activity of LCN2 in the vagina appears to be controlled by hormones, as treatment with topical estrogen concentrations led to an increase in LCN2 in vaginal douche samples of patients and in immortalized vaginal epithelial cell lines (72). All these findings shed light on the antibacterial activity of LCN2 in the female reproductive tract, which has previously been found in non-reproductive tissues (52).

### 4.5 Endometriosis

When endometrial-like tissue develops outside the uterine cavity, it is often associated with pelvic pain and infertility (73). This condition is known as endometriosis and often affects women in reproductive age (73). Nevertheless, the exact cause of this condition is not fully understood, but is associated with pro-inflammatory processes (73). Consistently, there is a significantly higher expression of serum LCN2 in patients with endometriosis compared to the control group (73). This increase was accompanied by increased quantities of the tumor marker cancer antigen 125 and the inflammatory C-reactive protein (74). In endometriosis patients, there was not only a systemic increase in LCN2, but also locally in endometrial cysts, as demonstrated by immunohistochemical staining (74). However, others did not find significant differences in serum LCN2 levels in endometriosis patients compared to the control group or between different stages of the disease, but the MMP9/LCN2 complex ratio was higher (75). Since endometriosis patients have an almost three times higher incidence of developing cancer, it is also likely that LCN2 is involved in the transition (76). Depending on their findings, Yamada and co-workers speculated that LCN2 is involved in the progression of ovarian carcinoma resulting from endometriosis (77).

Furthermore, a behavioral study showed that induced endometriosis in mice led to increased depression and is also reflected in the induction of the expression of various targets in the brain, including the induction of the LCN2 protein and mRNA in the amygdala in females with endometriosis (78). A study in mice that dealt with the molecular interactions that LCN2 plays in endometriosis found that LCN2 expression induces epithelial-to-mesenchymal transition (EMT) in stressed primary uterine endometrial cells promoting onset of endometriosis (79). More studies are essential to clarify how LCN2 is related to the initiation and progression of endometriosis. It would also be informative to investigate whether and how LCN2 fluctuates during the menstrual cycle, which has not been considered in existing studies and could possibly explain the differences seen.

## 5 LCN2 and cancers of the female reproductive system

Gynecological cancers can originate from various sites in the normal tissues of the female reproductive tract (80). LCN2 expression has been shown to be up-regulated in ovarian, cervical tissue, and endometrial cancer, as well as human ovarian cancer cell lines [https://www.proteinatlas.org/, last accessed on 21.3.2024].

### 5.1 Ovarian cancer

Hao and co-workers found an up-regulated expression of LCN2 in ovarian cancer patients, as well as in different ovarian cancer cell lines compared to normal tissue and cell lines (81). Using human ovarian cancer cell lines, the study provided evidence on the mechanism of LCN2 signaling ovarian cancer (81). LCN2 promotes tumor progression in ovarian cancer cells by activating the ERK/GSK3β/β-catenin signaling pathway (81).

Interestingly, LCN2 could be a suitable non-invasive biomarker for ovarian cancer as it is elevated not only in tissue, but also in serum and urine of patients (82–84). The expression of immunoreactive NGAL (irNGAL) in ovarian tumors changes with disease grade and this change is reflected in the concentration of NGAL/LCN2 in peripheral blood, which allows LCN2 to be used as a biomarker of tumor progression (83). Additionally, LCN2 has been detected among the most highly up-regulated genes in ovarian serous papillary carcinomas (85). Furthermore, LCN2 has been reported to increase intracellular iron levels in ovarian cancer cells but decrease oxidative stress, suggesting antioxidant capacity and allowing cancer cells to survive in stressful endometriotic cysts (77). Taken together, the data clearly demonstrate a positive correlation of LCN2 with tumor progression and targeting LCN2 or its signaling pathways could be a therapeutic strategy for targeting ovarian cancer.

### 5.2 Endometrial cancer

LCN2 mRNA and protein expression were strong in human patients with endometrial hyperplasia and, suggesting that it is involved in the tumorigenic process leading to endometrial carcinoma (86). Furthermore, there is a gradual increase in LCN2 in patients with endometrial hyperplasia and carcinoma (87). Consistent with this, LCN2 was found to be a component of the key signature in the onset of endometrial cancer in the glandular uterine epithelium, as its expression increased in precancerous atypical endometrial hyperplasia (88). A study with 85 high-grade endometrial cancer tissue samples showed strong LCN2 expression using immunohistochemical detection (88). Multivariate analysis revealed a positive correlation between LCN2 expression and shorter overall survival of these patients (89). Others found that increased LCN2 tissue expression is associated with aggressive characteristics and a poor prognosis in women with endometrial cancer (90). In line, immunohistochemical localization of LCN2 (as well as its receptor SLC22A17) was indicated LCN2 to be a prognostic factor in endometrial cancer (91). In endometrial cancer, there is not only a strong expression of LCN2 in the tissue, but also in the serum of the patients. In a study that included 123 women, out of whom 52 had endometrial cancer, significantly lower median serum levels of LCN2 were found in a group of patients with normal endometrium compared to the endometrial cancer group (92).

*In vitro* experiments confirmed the role of LCN2 in enhancing endometrial carcinoma cell survival and migration by mediating cytokine production (93). In addition, Lin and colleagues used the human endometrial carcinoma cell line RL95-2 to gain new insights into the LCN2 signaling mechanism and found that it is induced in a time-dependent manner by glucocorticoid stimulation (dexamethasone) and under starvation conditions (94). Furthermore, forced expression of LCN2 led to increased proliferation and invasion in the human endometrial cancer cell lines Ishikawa and HEC1B (87). A study by Miyamoto and co-workers provided information on signaling pathways of LCN2 in endometrial cancer (95). Their results showed that LCN2 promotes the survival of endometrial cancer cell lines via the PI3K pathway in a stressful environment and in an iron-dependent manner (95). Furthermore, others found that stimulation with LCN2 protein purified from mouse uterine fluid induced apoptosis in the RL95-2 endometrial cancer cell line (94). However, estrogen could not induce *Lcn2* expression in these cells (94). LCN2 expression is speculated to be involved in balance between cell death and proliferation in uterine tissue remodeling and is important in the progression of endometrial cancer.

### 5.3 Cervical cancer

Persistent infection with the human papilloma virus is a risk factor for the development of cervical cancer, including cervical adenomas and cervical squamous carcinomas (96). By analyzing 90 clinical specimens, it was found that elevated LCN2 tissue expression in cervical cancer is correlated with tumor metastasis and cancer progression (97). In addition, serum levels of LCN2 were also increased significantly patient with cervical cancer, suggesting it as a potential non-invasive biomarker for the diagnosis and prognosis of cervical cancer (97, 98). *In vitro* and *in vivo* studies in a xenograft model confirmed tumor-promoting role and provided evidence that LCN2 mediates cervical cancer through the EMT signaling pathway (97).

All mayor findings on LCN2 expression and role in female reproductive tract cancers have been summarized in a tabular form in **Table 3**.

**Table 3:** Expression levels of LCN2 in cancers of the human female reproductive tract.

Cancer type	Specimen/model	LCN2 expression and effect*	Reference
Endometrial	Cancerous tissue	↑	https://www.proteinatlas.org/, last accessed on 21.3.2024
	Endometrial hyperplasia, cancerous tissue	↑	(86, 87)
	Precancerous atypical endometrial hyperplasia tissue	↑	(88)
	Cancerous tissue	↑, correlation of LCN2 and shorter overall survival	(89)
	Cancerous tissue	↑, correlation of LCN2 expression with aggressive features and poor prognosis	(90)
	Cancerous tissue	↑, LCN2 can be used as prognostic marker	(91)
	Serum of cancer patient	↑, LCN2 can be used as non-invasive biomarker	(92)
	Carcinoma cell line (RL95-2)	LCN2 treatment promoted cancer cell survival and migration	(93)
	Carcinoma cell line (RL95-2)	Dexamethasone and starvation conditions upregulate LCN2	(94)
	Carcinoma cell line (HHUA, Ishikawa, HEC1A, HEC1B, KLE, RL-95-2	LCN2 overexpression increased cancer cell proliferation and invasion	(87)
	Carcinoma cell line (HHUA, HEC1A, HEC1B, KLE, RL95-2)	LCN2 overexpression promotes cancer cell survival in stressful environment	(95)
	Carcinoma cell line (RL95-2)	LCN2 isolated from murine uterine fluid induces apoptosis of human endometrial cancer cells	(94)
Ovarian	Cancerous tissue	↑	https://www.proteinatlas.org/, last accessed on 21.3.2024
	Carcinoma cell lines (OVCAR-5, SNU-8, RMG-I, OVKATE, EFO-21)	↑	https://www.proteinatlas.org/, last accessed on 21.3.2024
	Cancerous tissue, carcinoma cell lines (CAOV3, ES2, OVCAR3, SKOV3)	↑, LCN2 mediates tumor progression	(81)
	Cancerous tissue, serum, urine	↑, LCN2 can be used as non-invasive marker	(82–84)
	Ovarian serous papillary carcinomas	↑	(85)
	Ovarian clear cell carcinoma	↑, LCN2 promotes cancer cell survival	(77)
Cervical	Cancerous tissue	↑	https://www.proteinatlas.org/, last accessed on 21.3.2024
	Cancerous tissue, serum, cell lines (C33A)	↑, correlation of LCN2 with metastasis and cancer progression	(97)
	Serum of cancer patient	↑, LCN2 can be used as non-invasive marker	(98)

*↑ indicates an increased expression during the course of a specific pathology.

## 6 LCN2 and the pathology of the male reproductive tract

LCN2 is a known acute phase protein in non-reproductive tissues (52). Interestingly, a study examined *Lcn2* in the context of uropathogenic infections (99). In murine cauda epididymis, *Lcn2* mRNA was significantly increased after infection with uropathogenic *E. coli*, highlighting the antibacterial activity of LCN2 in reproductive tissues as seen in female uterine tissue (16, 99).

Male infertility can be caused by diseases of the testicles themselves or impaired testicular function, including defects in spermatogenesis, Leydig cell degeneration or poor sperm quality (100). One study from 2017, detected a strongly increased expression of *Lcn2* expression in the testes in mice as a consequence of induced infertility by busulfan treatment or bilateral cryptorchidism (35). Although the study did not examine molecular signaling mechanisms, the authors speculated that LCN2 is involved in germ cell apoptosis. Tanaka and colleagues conducted a study on the involvement of spermatogonia in testicular gene regulation. They discovered that germ cell-deficient mice completely lose the expression of certain genes, including *Lcn2* (32). Furthermore, they examined *Lcn2* expression in a juvenile spermatogonial depleted (*jsd/jsd*) mouse mutant, which has severe defects in spermatogonial differentiation (seminiferous tubules containing only type A spermatogonial germ cells and Sertoli cells), and in an artificial cryptorchid model (32). Interestingly, both models exhibited high expression of *Lcn2* in the testis, suggesting a potential relationship between *Lcn2* and germ cell apoptosis (32).

Kessel et al. analyzed LCN2 expression in testes from adult infertile *Esr1*-deficient mice (31). Interestingly, LCN2 protein expression was strongly increased in these animals compared with age-matched WT animals (31). These studies provide evidence that LCN2 has not only a physiological but also a pathophysiological role in the testes. However, it is mandatory to understand the underlying molecular mechanism, which likely involves estrogen receptor signaling.

Apart from its role in benign prostate diseases and the development and progression of prostate cancer (10, 101), LCN2 has hardly been studied at all in diseases of the male reproductive tract. In 2005, a study analyzed adult male germ cell tumors and found a high increase in LCN2 in teratomas without seminomas but not in other subtypes, classifying it as an suitable predictor (102).

For a more comprehensive understanding, it is also important to investigate the expression and localization of LCN2 in healthy human testes and in various diseases such as testicular cancer.

## 7 Conclusion

The presence of LCN2 has been confirmed in both male and female healthy reproductive systems by multiple studies. However, the available studies show that LCN2 has so far been studied much more intensively in a pathological context than in a physiological context. Nevertheless, we can speculate about the different functions of LCN2 in the female and male reproductive tract. Although it appears that in the female reproductive system LCN2 is involved in tissue reorganization during the menstrual cycle and pregnancy, the mechanism of its action and the cells responsible for its production have not yet been determined. However, it has been shown to serve as a component of the maturation and motility component of sperm in male reproductive organs. Furthermore, several studies report the deregulation of LCN2 in numerous reproductive tissue pathologies, including fertility defects. Considering that the literature overview provides clear evidence that LCN2 is a key component in fertility, and that no information on the actual mechanisms of its action is currently available, further research on this topic would be beneficial to understand its precise function in the reproductive system, as well as its therapeutic potential.

## Author contributions

MK: Conceptualization, Data curation, Writing – original draft, Writing – review & editing. PBMS: Data curation, Visualization, Writing – review & editing. RW: Data curation, Funding acquisition, Supervision, Writing – review & editing. SKS: Conceptualization, Data curation, Supervision, Writing – original draft, Writing – review & editing.
